# Women’s Cardiovascular Health: Prioritizing the Majority Minority

**DOI:** 10.3390/jcdd10030128

**Published:** 2023-03-17

**Authors:** Anna E. Bortnick, Edita Pllana, Diana S. Wolfe, Cynthia C. Taub

**Affiliations:** 1 Department of Medicine, Division of Cardiology, Montefiore Medical Center, Albert Einstein College of Medicine, New York, NY 10467, USA; 2 Maternal Fetal Medicine-Cardiology Joint Program, Montefiore Medical Center, Albert Einstein College of Medicine, Bronx, NY 10461, USA; 3 Section of Cardiovascular Medicine, Heart and Vascular Center, Dartmouth-Hitchcock Medical Center, Geisel School of Medicine at Dartmouth, Lebanon, NH 03756, USA; 4 Department of Electrophysiology, Clinic of Cardiology, University Clinical Center of Kosovo, 10000 Prishina, Kosovo; 5 Department of Obstetrics and Gynecology and Women’s Health, Division of Maternal Fetal Medicine, Montefiore Medical Center, Albert Einstein College of Medicine, Bronx, NY 10467, USA

Women make up the majority of the global population, and are burdened by cardiovascular disease as their most common cause of death [1]. However, women are in the minority of individuals receiving cardiovascular interventions, advanced heart failure therapy and guidelines-based medical therapy for cardiovascular disorders, even in an era of robust diagnostic technology and well-proven therapeutic approaches [2,3]. In this Special Issue of the journal, authors from Europe, Asia, Australia and North America address under-treated and under-researched cardiovascular health concerns unique to women (a summary of some highlights can be found in Table 1). Most address the increased cardiovascular risk in pregnancy, as there has been an alarming increase in maternal deaths in the past two decades in the US, especially among Black and Native American or Alaskan women [4]. Pregnancy is a natural yet powerful stressor of the cardiovascular system, which can unmask or provoke a cardiac condition, and decision-making in these cases can be complex [5]. The broad themes in this Special Issue include cardiovascular disease (CVD) screening, congestive heart failure (CHF), and acute coronary syndromes (ACS).

CVD screening may take the form of risk prediction for gestational diabetes mellitus and hypertensive disorders, common complications of pregnancy. A combination of demographics, clinical characteristics and vital signs appears to be useful for screening those at risk for cardiovascular decompensation peripartum, with a new CVD algorithm and toolkit that can be built into routine visits [6]. Additionally, echocardiography, even in low-risk individuals and especially in those who have hypertension or obesity, may reveal abnormal findings which could be important in risk stratification or treatment [7]. Lastly, a point-of-care ultrasound of the heart and lungs on the Labor and Delivery ward affords the quicker triaging of cardiac versus non-cardiac diagnoses, such as acute congestive heart failure [8].

CHF is a leading cause of morbidity and mortality in pregnant women in the US [4]. Therapy should be optimized for those with preexisting CHF and acute CHF, and they should be treated and monitored closely. There is an increased risk of acute CHF in survivors of childhood or adolescent chemoradiation, which can complicate pregnancy. With more survivors of cancer and congenital heart disease attaining reproductive age, CHF is becoming a complicating factor for pregnancy in these individuals [9]. Arrhythmia is common, and rising due to increased maternal age and increases in hypertension, diabetes mellitus and obesity. In CHF and arrhythmia, cardioselective beta blockers and diuretics are a mainstay of treatment, provided that the patient is not in cardiogenic shock [10]. Black individuals experience peripartum cardiomyopathy (PPCM) more frequently than their white or Hispanic/Latina counterparts [11]. Individuals with PPCM may need short-term mechanical support until recovery, or as a bridge to left ventricular assist devices or heart transplant. Socioeconomic disadvantage is a compounding factor, in those who progress to advanced heart failure therapies and for global access to ACS care. PAH, either idiopathic or due to chronic thromboembolic disease, is more frequent in women than men [12,13]. PAH hemodynamically stresses the right ventricle, and pregnancy can lead to RV failure and death, which is a reason for pregnancy avoidance or termination. In rare cases, and with subspeciality management, as described in this Special Issue, there are some individuals who have delivered successfully [14,15].

ACS presentation can be due to microvascular disease or plaque rupture in women [16]. Spontaneous coronary artery dissection is a particular morbid form of ACS associated with arteriopathies, and is most common late in pregnancy or postpartum [17,18]. Unstable patients with ACS should undergo cardiac catheterization, but many barriers to care exist that limit women’s access.

**Table 1:** A summary of the key points, discussions and implications of publications in this Special Issue.

Authors	Key Points	Discussion and Implications
Hennessey and Taub et al. [7]	**Background**: ECHOs are performed in pregnancy for suspected cardiac disease **Study Design**: Retrospective cohort study **Results**: Low risk individuals with abnormal ECHO were more likely to have CD or PTD	Abnormal ECHO among low-risk pregnant persons is associated with adverse obstetric outcomes
Wu and Hua et al. [13]	**Background**: Women have worse outcomes in CTEPH. The reasons are multifactorial **Study Design**: Prospective cohort study of CTEPH 2013–2019 **Results**: Revascularization, therapy and correction of anemia improved clinical outcomes	Understanding sex-specific features of CTEPH may improve outcomes
Wolfe and Bortnick et al. [11]	**Background**: PPCM is a major cause of maternal morbidity and mortality **Study Design**: Retrospective, age- and race-matched case–control 1999–2015 **Results**: Individuals with PPCM had worse SES and baseline health	Addressing social determinants of health is important for PPCM outcomes
Vaidya and Forfia et al. [14]	**Background**: PAH in pregnancy has been associated with maternal morbidity and mortality; new medications and multidisciplinary management may improve outcomes **Study Design**: A single center retrospective observational study between 2013–2021 **Results**: Reported successful maternal and fetal outcomes	Pregnant patients with PAH may have good outcomes when managed by a specialized center
Kwampong and Sharma et al. [5]	**Background**: Pregnancy outcomes in moderate hyperglycemia have not been studied **Study Design**: Retrospective comparative review of the Boston Birth Cohort 1998–2016 **Results**: Second and third tertiles of serum glucose had higher birth weight and more hypertension	Moderately hyperglycemic patients may be at higher risk for adverse outcomes
Negrea and Pop et al. [16]	**Background**: Atypical presentations of acute coronary syndromes can delay treatment **Study Design**: Retrospective review of unstable angina and NSTEMI from 2014 to 2015 **Results**: Women were more hypertensive with microvascular disease	Sex-specific atypical presentations in ACS need to be considered
Bansal and Zhang et al. [9]	**Background**: Cancer survivors are achieving reproductive age **Study Design**: Review of risk factors in cancer survivors who seek pregnancy **Results**: Cardiotoxicity prior to pregnancy portends higher risk for CHF pre- or postpartum	Cancer survivors should be screened for cardiotoxicity prior to pregnancy
Cader and Gulati et al. [9]	**Background**: There are sex differences in presentation and access in ACS globally **Study Design**: Review **Results**: Sex disparities in ACS management compounded by SES, education, and access	Global sex-related barriers to ACS care include access, local practice, cultural differences, SES, education
Tamirisa and Volgman et al. [10]	**Background**: Arrhythmias and CHF co-exist in pregnancy **Study Design**: Review **Results**: AVNRT, AVRT more common with structurally normal heart. AFIB is increasing	Treatment pre-conception is recommended with known arrythmia
O’Kelly and Wood et al. [17]	**Background**: MI during pregnancy is more common in the third trimester and early post-partum **Study Design**: Review **Results**: SCAD is most common. Imaging is necessary to evaluate for fibromuscular dysplasia	In low-risk NSTEMI, consider medical management
Daraz and Wolfe et al. [15]	**Background**: Idiopathic PAH portends high maternal and fetal mortality in pregnancy **Study Design**: Review **Results**: Adaptive changes during pregnancy can adversely affect the RV	Delivery should be at a specialized center with possibility for VA-ECMO
Chambers and Hameed et al. [6]	**Background**: Maternal mortality is rising in the US **Study Design**: Review **Results**: Universal CVD screening may identify risks, decrease delays and inequities	ACOG endorses universal CVD screening antepartum and postpartum
Thong and Enticott et al. [19]	**Background**: Prediction for GDM and HDP is important for avoiding adverse outcomes **Study Design**: Review **Results**: Biomarkers, clinical risk factors improve accuracy of prediction for preeclampsia	Novel biomarkers improved risk prediction for preeclampsia
Thachil and Sokol et al. [12]	**Background**: Sex-specific outcomes of pulmonary embolism are understudied **Study Design**: Systematic review **Results**: Women with PE tended to be older, with dyspnea, and higher NT-proBNP vs. men	PE mortality was higher in women
Algodi and Taub et al. [8]	**Background**: POCUS is useful for heart and lung examination **Study Design**: Review **Results**: POCUS is useful for cardiac conditions and pulmonary edema in pregnancy	Providers should be trained to perform POCUS on L&D
Mantovani and Guiducci et al. [18]	**Background**: SCAD is exceedingly rare in the second trimester of pregnancy **Study Design**: Case Report **Results**: SCAD in the second trimester successfully treated with PCI	Catheterization recommended for unstable SCAD

ACOG = American College of Obstetrics and Gynecology; ACS = acute coronary syndromes; AVNRT = atrioventricular nodal reentry tachycardia; AVRT = atrioventricular reentry tachycardia; CD = C-section delivery; CHF = congestive heart failure; CTEPH = chronic thromboembolic pulmonary hypertension; CVD = cardiovascular; ECHO = echocardiogram; GDM = gestational diabetes mellitus; HDP = hypertensive disorders of pregnancy; L&D = labor and delivery; MI = myocardial infarction; NT-proBNP = N-terminal pro beta natriuretic peptide; NSTEMI = non-ST elevation myocardial infarction; PAH = pulmonary arterial hypertension; PE = pulmonary embolism; POCUS= Point-of-care ultrasonography; PPCM = peripartum cardiomyopathy; PTD = pre-term delivery; RV = right ventricle; SCAD = spontaneous coronary artery dissection; SES = socioeconomic status; VA-ECMO = venous-arterial extracorporeal membrane oxygenation.

As we can see from this Special Issue, specialized care is needed for women due to their unique cardiovascular risk [19]. Knowledge gaps need to be filled to better understand sex-specific pathophysiology and sex-disaggregated data, and to optimize individualized treatment options. Multidisciplinary specialized centers and programs are one way to work towards improving outcomes. Developing new sex-specific approaches, while also delivering proven standard of care to women, is key, until this discrepancy regarding the beneficiaries of modern cardiovascular treatment is resolved.

## References

[1] Naghavi M., Abajobir A.A., Abbafati C., Abbas K.M., Abd-Allah F., Abera S.F., Aboyans V., Adetokunboh O., Afshin A., Agrawal A. (2017). Global, Regional, and National Age-Sex Specific Mortality for 264 Causes of Death, 1980–2016: A Systematic Analysis for the Global Burden of Disease Study 2016. Lancet.

[2] McSweeney J.C., Rosenfeld A.G., Abel W.M., Braun L.T., Burke L.E., Daugherty S.L., Fletcher G.F., Gulati M., Mehta L.S., Pettey C. (2016). Preventing and Experiencing Ischemic Heart Disease as a Woman: State of the Science. Circulation.

[3] Cader F.A., Banerjee S., Gulati M. (2022). Sex Differences in Acute Coronary Syndromes: A Global Perspective. J. Cardiovasc. Dev. Dis..

[4] Petersen E.E. (2019). Vital Signs: Pregnancy-Related Deaths, United States, 2011–2015, and Strategies for Prevention, 13 States, 2013–2017. MMWR Morb. Mortal. Wkly. Rep..

[5] Kwapong Y.A., Boakye E., Wang G., Hong X., Lewey J., Mamas M.A., Wu P., Blaha M.J., Nasir K., Hays A.G. (2022). Maternal Glycemic Spectrum and Adverse Pregnancy and Perinatal Outcomes in a Multiracial US Cohort. J. Cardiovasc. Dev. Dis..

[6] Chambers M.E., De Zoysa M.Y., Hameed A.B. (2022). Screening for Cardiovascular Disease in Pregnancy: Is There a Need?. J. Cardiovasc. Dev. Dis..

[7] Hennessey K.C., Ali T.S., Choi E., Ortengren A.R., Hickerson L.C., Lee J.M., Taub C.C. (2022). Association between Abnormal Echocardiography and Adverse Obstetric Outcomes in Low-Risk Pregnant Women. J. Cardiovasc. Dev. Dis..

[8] Algodi M., Wolfe D.S., Taub C.C. (2022). The Utility of Maternal Point of Care Ultrasound on Labor and Delivery Wards. J. Cardiovasc. Dev. Dis..

[9] Bansal N., Hazim C.F., Badillo S., Shyam S., Wolfe D., Bortnick A.E., Garcia M.J., Rodriguez C.J., Zhang L. (2022). Maternal Cardiovascular Outcomes of Pregnancy in Childhood, Adolescent, and Young Adult Cancer Survivors. J. Cardiovasc. Dev. Dis..

[10] Tamirisa K.P., Dye C., Bond R.M., Hollier L.M., Marinescu K., Vaseghi M., Russo A.M., Gulati M., Volgman A.S. (2022). Arrhythmias and Heart Failure in Pregnancy: A Dialogue on Multidisciplinary Collaboration. J. Cardiovasc. Dev. Dis..

[11] Wolfe D.S., Liu C., Alboucai J., Karten A., Mushi J., Yellin S., Berkowitz J.L., Vega S., Felix N., Liaqat W. (2022). Maternal Outcomes in Women with Peripartum Cardiomyopathy versus Age and Race-Matched Peers in an Urban US Community. J. Cardiovasc. Dev. Dis..

[12] Thachil R., Nagraj S., Kharawala A., Sokol S.I. (2022). Pulmonary Embolism in Women: A Systematic Review of the Current Literature. J. Cardiovasc. Dev. Dis..

[13] Wu Y., Hu S., Yan X.-X., Peng F.-H., Tan J.-S., Guo T.-T., Gao X., Hua L. (2022). Chronic Thromboembolic Pulmonary Hypertension in Females: Clinical Features and Survival. J. Cardiovasc. Dev. Dis..

[14] Vaidya A., Oliveros E., Mulla W., Feinstein D., Hart L., Forfia P. (2022). Management of Pulmonary Arterial Hypertension in Pregnancy: Experience from a Nationally Accredited Center. J. Cardiovasc. Dev. Dis..

[15] Daraz Y., Murthy S., Wolfe D. (2022). Pregnancy in Pulmonary Arterial Hypertension: A Multidisciplinary Approach. J. Cardiovasc. Dev. Dis..

[16] Negrea M.O., Zdrenghea D., Teodoru M., Neamțu B., Cipăian C.R., Pop D. (2022). Gender Particularities and Prevalence of Atypical Clinical Presentation in Non-ST Elevation Acute Coronary Syndrome. J. Cardiovasc. Dev. Dis..

[17] O’Kelly A.C., Ludmir J., Wood M.J. (2022). Acute Coronary Syndrome in Pregnancy and the Post-Partum Period. J. Cardiovasc. Dev. Dis..

[18] Mantovani F., Navazio A., Tortorella G., Guiducci V. (2022). Unique Case of Spontaneous Left Main Coronary Dissection in Second Trimester of Pregnancy Successfully Treated with Percutaneous Coronary Intervention: A Happy Ending. J. Cardiovasc. Dev. Dis..

[19] Thong E.P., Ghelani D.P., Manoleehakul P., Yesmin A., Slater K., Taylor R., Collins C., Hutchesson M., Lim S.S., Teede H.J. (2022). Optimising Cardiometabolic Risk Factors in Pregnancy: A Review of Risk Prediction Models Targeting Gestational Diabetes and Hypertensive Disorders. J. Cardiovasc. Dev. Dis..

